# Pulsed Electric Field as an Alternative Pre-treatment for Drying to Enhance Polyphenol Extraction from Fresh Tea Leaves

**DOI:** 10.1007/s11947-018-2199-x

**Published:** 2018-11-01

**Authors:** Zhibin Liu, Erik Esveld, Jean-Paul Vincken, Marieke E. Bruins

**Affiliations:** 1 Laboratory of Food Chemistry, Wageningen University, P.O. Box 17, 6700 AA Wageningen, The Netherlands; 2 Institute of Food Science & Technology, Fuzhou University, Fuzhou, 350108 People’s Republic of China; 3 Food & Biobased Research, Wageningen University & Research Centre, PO Box 17, 6700 AA Wageningen, The Netherlands

**Keywords:** Pulsed electric field, Polyphenol extraction, Fresh tea leaves, Phenolic profile, Scanning electron microscope

## Abstract

Drying is an essential pre-treatment prior to extraction of tea polyphenols from tea leaves, which is a time and energy-intensive process. In this study, pulsed electric field (PEF) was utilized to replace the conventional thermal dehydration procedure before the phenolic extraction. The influence of different PEF conditions on total polyphenol yield from fresh tea leaves combined with a solid-liquid extraction were compared. PEF treatment at 1.00 kV/cm electric field strength, 100 pulses of 100 μs pulse duration, and 5 s pulse repetition, which delivered 22 kJ/kg and induced 1.5 °C of temperature increase, was used for further study on the extraction kinetics of green tea catechins. The results indicated that compared to oven drying, PEF pre-treatment increased the extraction rate by approximately two times, without significantly altering the phenolic profiles, as revealed by using liquid chromatography combined with mass spectrometry. Scanning electron microscopy imaging revealed that PEF pre-treatment induced the formation of inhomogeneously distributed pores and protuberances on the surface of leaf tissues, which might facilitate the penetration of extraction solvent and the migration of phenolics. This study demonstrates that PEF as a time and energy efficient processing method is a promising alternative for the conventional drying process before further tea polyphenol extraction.

## Introduction

It has been widely accepted that tea, derived from the leaves of *Camellia sinensis*, is a healthy beverage. The pharmaceutical benefits of tea are generally attributed to its phenolic compounds, because of their anti-oxidative, anti-inflammatory, anti-microbial, anti-tumor, and anti-aging properties (da Silva Pinto 2013; Bansal et al. 2013). Extracted tea polyphenols are widely used in various applications like functional beverages, functional food, dietary supplements, and cosmetics. Increasing demand for tea polyphenols in food and healthcare applications has boosted the growth of the tea polyphenol market. Extraction of polyphenols from tea leaves into a concentrated form is attracting considerable attention of industry.

With regard to the extraction of phytochemicals from plant-based materials, two primary processes are required: (i) cell wall and cell membrane disruption and (ii) intracellular substance migration. Mechanical pressing or grinding is widely used to completely disrupt the cell, but it results in non-selective release of all cell components and creates large amounts of cell debris, which complicates the subsequent fractionation (Postma et al. 2016). Drying is another widely used procedure for disruption of plant cells. This procedure causes breakage and collapse of cell walls and the formation of large cavities and intercellular spaces, consequently allowing the cellular substances to be easily extracted (Drosou et al. 2015). However, the conventional thermal dehydration procedure is considered to be time and energy intensive. Moreover, drying may also reduce the bioactivities of thermally instable constituents. For intracellular substance migration, aqueous and organic solvent extraction are the primary methods (Azmir et al. 2013). In tea polyphenol extraction, thermal dehydration of tea leaves followed by organic solvent extraction is the most widely used method (Pasrija and Anandharamakrishnan 2015). The organic solvent can be evaporated to obtain the pure tea polyphenols. In addition to these conventional extraction methods, different processes including supercritical fluid extraction, microwave-assisted extraction, ultrasonic-assisted extraction, and pulsed electric field-assisted extraction can be employed to extract phenolics from tea leaves (Pasrija and Anandharamakrishnan 2015). Different extraction techniques have various impacts on the rate, yield, purity, and composition of final products. Moreover, the time and energy input are also different.

Pulsed electric field (PEF) is an emerging technology for processing cells by means of brief pulses of a strong electric field (Jeyamkondan et al. 1999). Applying an external electric field to the cells results in pore formation in the membrane of plant cells, facilitating the release of the cellular contents, such as phenolic components (Donsì et al. 2010). This permeabilization of cell membranes can be achieved at moderate electric fields (< 10 kV/cm) and low specific energies (< 10 kJ/kg), and thus is a non-thermal and energy efficient technique of extraction (Puértolas et al. 2013). Donsì et al. (2010) and Yan et al. (2017) reviewed the applications of this technique for extraction of bioactive compounds from various food matrices and illustrated its efficiency. Tea polyphenol extraction with PEF has only been reported once (Zderic and Zondervan 2016). However, with fresh tea leaves as material and water as extraction solvent, the highest recovery of polyphenols reported in this study was only 27% (extracted polyphenol / total polyphenol content in fresh tea leaves). As the epicuticular waxes existing on the surface of the leaves can act as a barrier to extraction, the efficiency of aqueous extraction with PEF is relatively low. However, the same study showed that electroporation through PEF treatment had potential for cell rupture of fresh tea leaves to replace the time and energy-intensive dehydration step. Therefore, there is still significant room for the improvement of extraction yield, for example, utilizing a combination of PEF pre-treatment and organic solvent extraction may achieve higher yield. In addition, besides the electroporation effect of PEF treatment, other effects, such as the alteration of phenolic profile and ultrastructural morphology, are not previously discussed in current literatures.

In the present study, PEF pre-treatment was combined with organic solvent extraction for tea polyphenols from fresh tea leaves. The extraction yield, catechin extraction kinetics, and phenolic profiles of extracts were evaluated by using Folin-Ciocalteu assay and liquid chromatography combined with mass spectrometry (LC/MS). Furthermore, the changes in leaf morphology induced by PEF were further assayed by using scanning electron microscopy (SEM) for the first time.

## Materials and Methods

### Fresh Tea Leaves and Other Chemicals

Fresh tea leaves (*Camellia sinensis*) were collected from tea plants grown in the Netherlands. Immediately following the collection, leaves were transferred to the laboratory and washed with distilled water for further treatment.

Gallic acid and Folin-Ciocalteu’s reagent were purchased from Sigma-Aldrich (St. Louis, MO, USA). Pure acetone, methanol, and ethanol were purchased from Merck (Darmstadt, Germany). Standards of epicatechin (EC), epicatechin gallate (ECG), epigallocatechin (EGC), and epigallocatechin gallate (EGCG) were purchased from Sigma-Aldrich (St. Louis, MO, USA). Ultrahigh-performance liquid chromatography/mass spectrometry (UHPLC/MS)-grade acetonitrile with 0.1% (*v*/*v*) formic acid and water with 0.1% (*v*/*v*) formic acid were purchased from Biosolve (Valkenswaard, The Netherlands). Water was prepared using a Milli-Q water purification system (Millipore, Billerica, MA, USA).

### PEF Pre-treatment

**Fig. 1:**
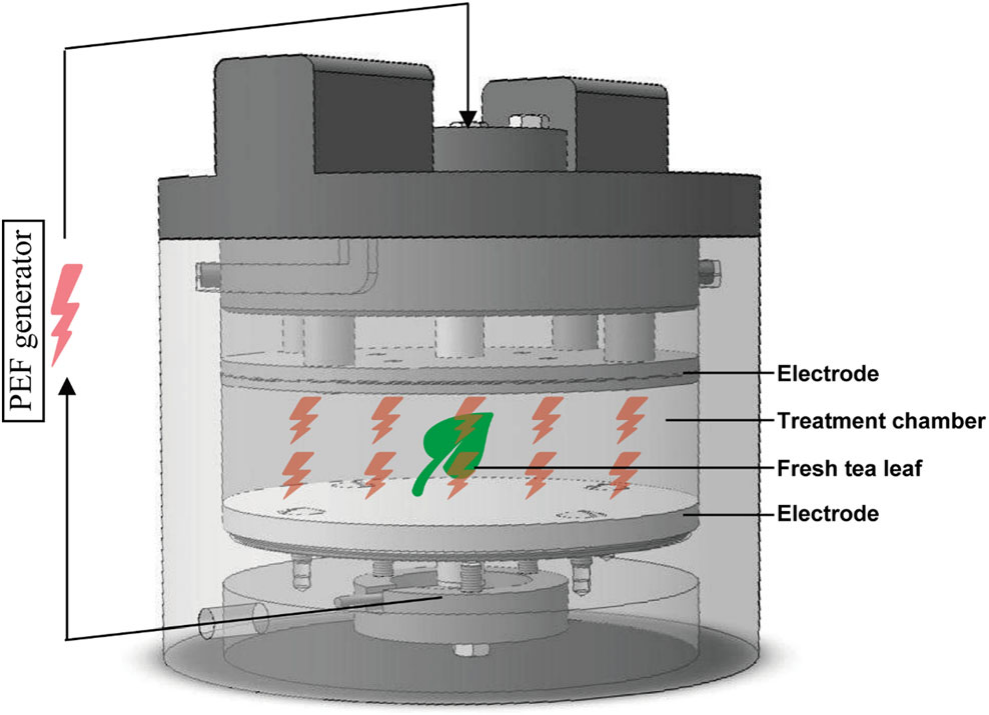
Schematic diagram of PEF chamber

PEF pre-treatments were conducted using a batch PEF system (NP110-60, IXL Netherlands b.v.) at room temperature (ca. 20 °C), which consisted of a high-voltage pulse generator (50 μF capacitance) and a PEF treatment chamber, as shown in Fig. 1. The distance between the 5-cm semi-diameter (*r*) parallel stainless steel electrodes was set to 2 cm (*d*). The voltage (*U*) and current (*I*) during the pulse over the treatment chamber were recorded via a high-voltage probe and peak current sensor on a digital oscilloscope (DS1054Z, Rigol, USA). The conductivity (σ) was calculated as: $\upsigma =\left(\frac{I}{U}\right)\bullet \left(\frac{d}{pi\bullet {r}^2}\right)$. The temperature elevation and electrical conductivity change of the solution in PEF chamber before and after PEF treatment were recorded.

One piece of whole fresh tea leaf (200–400 mg) was introduced into the chamber with 200 mL of 1.86 g/L KCl solution (Zderic and Zondervan 2016) and 1.0 g/L ascorbic acid to prevent browning by tea polyphenols. Subsequently, samples were subjected to various PEF treatment conditions. The monopolar pulses of near rectangular shape were delivered resulting in an electric field strength of *E* = 0.75 kV/cm, 1.00 kV/cm, or 1.25 kV/cm. The pulse duration *τ* = 100 μs and pulse repetition time △*t* = 5 s were fixed and the number of pulses was varied from *n* = 10, 50, 100, to 200. The total PEF treatment time (*t*) refers to the product *n* ∙ *τ*. The specific energy input (*Q*) is calculated as: *Q* = (*U* ∙ *I* ∙ *t*)/*m* (Raso et al. 2016; Grimi et al. 2007), where *U* is the voltage, *I* is the electric current strength, *t* is total treatment time of PEF, and *m* is the total mass of water-sample mixture exposed to the PEF pulses.

### Acetone Extraction

After the PEF pre-treatment, the tea leaves were transferred into a beaker for further organic solvent extraction. A 50% acetone/water (*w*/*w*) solution with a biomass to solvent ratio of 1:100 was used for extraction (Turkmen et al. 2006). The extraction was operated at room temperature with stirring at 250 rpm (Variomag multipoint magnetic stirrer HP 15, Oberschleißheim, Germany). For optimization of the PEF treatment conditions, the samples extracted for 2 h were compared on the basis of total polyphenol content, assayed with Folin-Ciocalteu’s reagent. Extraction kinetics were studied during 24 h under optimized PEF treatment conditions. During the extraction, at selected time points (0 min, 10 min, 0.5 h, 1 h, 1.5 h, 2 h, 4 h and 24 h), 200 μL of extract was collected for the determination of the concentration of the four native green tea catechins, including EC, EGC, ECG, and EGCG, by using LC/MS. These four catechins have been reported to be the main native tea phenolics (Balentine et al. 1997). The phenolic profiles of the extract at 2 h were further analyzed by LC/MS. Fresh (untreated) and oven dried (80 °C for 12 h) tea leaves were also extracted with the same procedure for comparison.

### Total Polyphenol Analysis

The total amount of phenolics of the extracts was determined using the Folin-Ciocalteu method (Obanda et al. 1997). Briefly, 1.0 mL sample, 5.0 mL Folin-Ciocalteu’s reagent (diluted 10 times), and 4 mL sodium carbonate (7.5% *w*/*v*) were introduced into test tubes. After 60 min, the absorbance at 765 nm was measured. Gallic acid (concentration ranging from 0.02 to 0.1 mg/mL) was used as an external standard.

### Native Catechin Content and Phenolic Profile Analysis

The native catechin content and phenolic profile analysis were performed on an Accela UHPLC system (Thermo Scientific, San Jose, CA, USA) equipped with a binary pump, an autosampler at 15 °C, and a diode array detector. An Acquity UHPLC BEH C18 column (150 mm × 2.1 mm, 1.7 μm; Waters, Milford, MA) with a VanGuard guard column (5 mm × 2.1 mm, 1.7 μm; Waters, Milford, MA) was used to perform the chromatographic separation. The injection volume was 1.0 μL. The flow rate was 400 μL/min at a column temperature of 20 °C. Eluents used were water (A) and acetonitrile (B), both containing 0.1% (*v*/*v*) formic acid. The elution program was started by running isocratically at 1% B for 1.0 min, followed by 1.0–3.0 min linear gradient to 15% B, 3.0–15.0 min linear gradient to 50% B, 15.0–17.0 min linear gradient to 99% B, 17.0–18.0 min linear gradient to 1% B, and 18.0–21.0 min isocratic at 1% B. The eluent was adjusted to its starting composition in 1 min, followed by equilibration for 7 min. Detection wavelengths for ultraviolet-visible (UV-vis) were set to the range of 200–600 nm. Data were recorded at 20 Hz.

After chromatographic separation, a Velos Pro linear ion trap mass spectrometer (Thermo Scientific, San Jose, CA, USA) equipped with a heated ESI probe coupled to the UHPLC system was used for analysis. Nitrogen was used as sheath gas and auxiliary gas. Data were collected in negative ionization mode over the *m/z* range of 150–1500. Data-dependent MS^2^ analysis was performed with a normalized collision energy of 35%. The MS^2^ fragmentation was performed on the most intense product ion in the MS spectrum, with a dynamic exclusion for 5 s. Most settings were optimized via automatic tuning using LTQ Tune Plus 2.7 (Thermo Scientific, San Jose, CA, USA). The transfer tube temperature was 350 °C, and the source voltage was 4.0 kV. Data acquisition and reprocessing were performed with Xcalibur 2.2 (Thermo Scientific, San Jose, CA, USA). Contents of the four primary catechins, including EC, EGC, ECG, and EGCG, in each sample were calculated by using external standards of EC, EGC, ECG, and EGCG.

### Micromorphology Imaging

The micromorphology of PEF treated, fresh, and oven-dried tea leaves were analyzed by SEM. Pieces of leaf tissues (10 × 20 mm) were cut with a blade from the middle of the leaf avoiding the central vein and fixed overnight in 100% methanol. Before further treatment, the pieces were cut to smaller pieces (about 10 × 10 mm) and brought into a 50/50 mixture of methanol/ethanol followed by 100% ethanol (Talbot and White 2013). Hereafter, the samples were critical point dried with carbon dioxide (EM CPD 300, Leica, Vienna, Austria), attached on SEM sample holders using carbon adhesive tabs (EMS, WA, USA) and sputter coated with 15 nm tungsten (EM SCD 500, Leica, Vienna, Austria). The upper side of the leaves was analyzed with a field emission scanning electron microscope (Magellan 400, FEI, Eindhoven, The Netherlands) with SE detection at 2 kV and 6.3 pA.

### Statistical Analysis

All data were expressed as means ± standard deviations (SD) of triplicate measurements. The differences between various treatments were analyzed by Duncan’s multiple range test using SPSS software (Version 19.0.0, IBM, USA), while the significance threshold was established at 0.05.

## Results and Discussion

### PEF Conditions

**Fig. 2:**
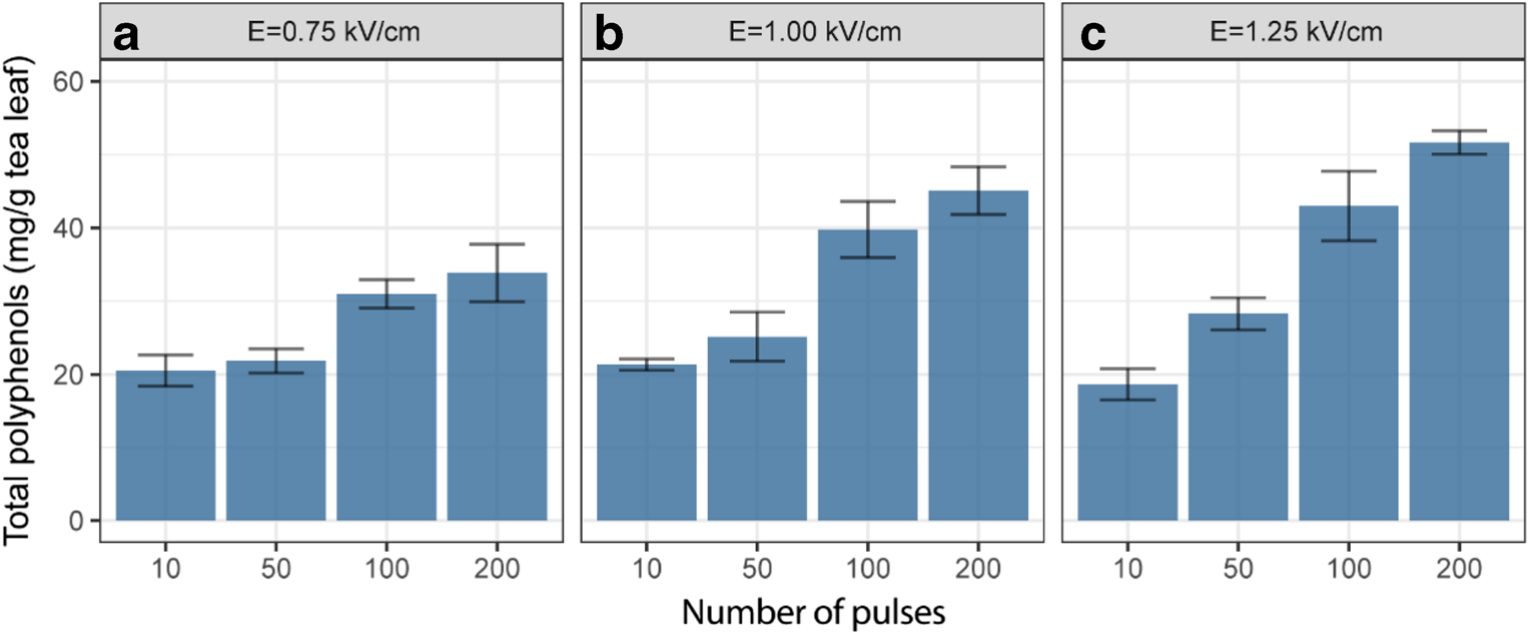
**a**–**c** The total polyphenols extracted from fresh tea leaves pre-treated with PEF at different electric field strength and number of pulses. Values represent the means ± SD of triplicate measurements

**Fig. 3:**
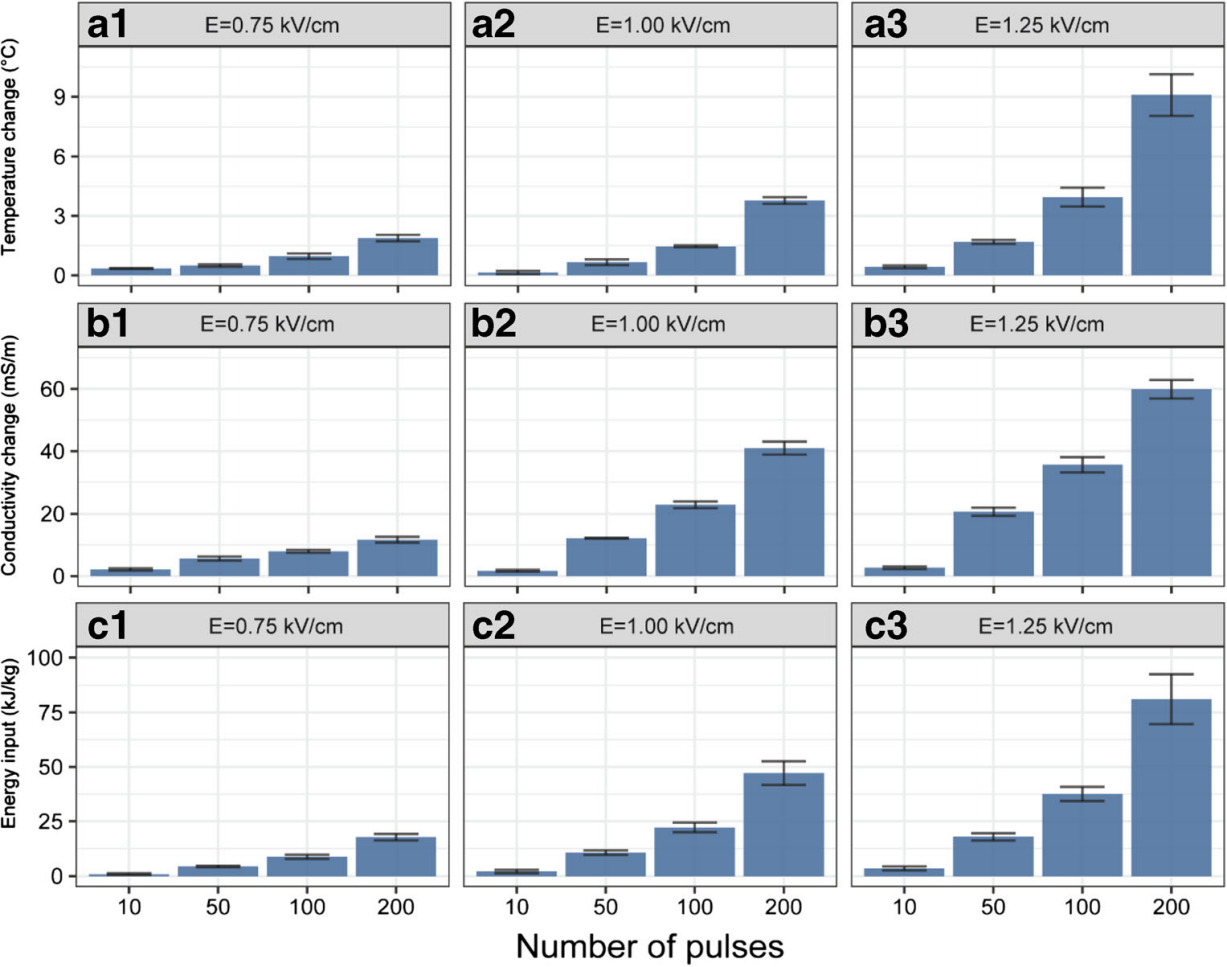
The temperature change (A1–A3), conductivity change (B1–B3), and specific energy input (C1–C3) during the PEF treatment at different electric field strength and number of pulses. Values represent the means ± SD of triplicate measurements

Many studies have shown the efficiency of the application of PEF as pre-treatment combined with organic solvent extraction for bioactive compound transfer from plant matrices (Zbinden et al. 2013; Boussetta et al. 2014; Jaeschke et al. 2016). In the present study, various PEF pre-treatment conditions were used on the fresh tea leaves, followed by a 2-h extraction with 50% acetone/water (*w*/*w*). Turkmen et al. have highlighted the efficiency of a mixture of acetone and water (1:1) for tea polyphenol extraction (Turkmen et al. 2006), and this was accordingly chosen as the extraction solvent in this study. In order to take more samples in time during extraction without disturbing the experiment, a relatively high solid to liquid ratio (1:100) was used. The yields of total polyphenols (based on fresh weight of leaves) resulting from these PEF conditions are compared in Fig. 2. Their temperature elevation, solution conductivity change, and specified energy input are presented in Fig. 3. With the increase of electric field intensity and pulse number, higher recovery of total polyphenols was observed. The highest yields are obtained at the most intense treatment condition tested (1.25 kV/cm, 200 pulses), which also resulted in the highest conductivity changes. This increment of solution conductivity relates to the release of intracellular ions, which can be interpreted as the extent of cell disruption (Fincan et al. 2004). Both the field strength and number of pulsed can be used to increase polyphenol extraction for all polyphenols tested. The most extreme condition used an energy consumption of 81 kJ/kg and led to a temperature increment of 9.1 °C. Under thermally isolated conditions, this would be maximally 20 °C given the specific energy input. It was reported that the specific energy consumption for convective drying of grapes was ca. 81 MJ/kg (Tulasidas et al. 1995). Compared with this, the 1000 times lower energy consumption of 81 kJ/kg used in the PEF pre-treatment is negligible. However, the temperature increment of 9.1 °C was undesirable.

Following the most intense treatment condition, the samples exposed to the next three PEF conditions (1.25 kV/cm, 100 pulses; 1.00 kV/cm, 200 pulses; and 1.00 kV/cm, 100 pulses) had similar tea polyphenol yields (*p* > 0.05), which were significantly higher than those from the rest samples (*p* < 0.05). Under these three PEF conditions, the energy consumption and temperature elevation were significantly lower than the most extreme condition (*p* < 0.05). Thus, for further testing of the kinetics of polyphenol extraction and the comparison to drying as a pre-treatment, a field strength of 1.00 kV/cm and 100 pulses was chosen. Here, the energy consumption and temperature elevation were 22 kJ/kg and 1.5 °C, respectively. These PEF conditions enabled subsequent extraction of 398 mg/L tea polyphenols in 2 h, which accounted for approximately 77% of total polyphenols (based on a 24-h acetone extraction). In absence of PEF treatment, under the same 2-h acetone extraction, only approximately 37% of total polyphenols were extracted from fresh tea leaves. The substantial increase in extraction recovery implied the improvement of permeability of cell membranes after PEF treatment.

### Extraction Kinetics

**Fig. 4:**
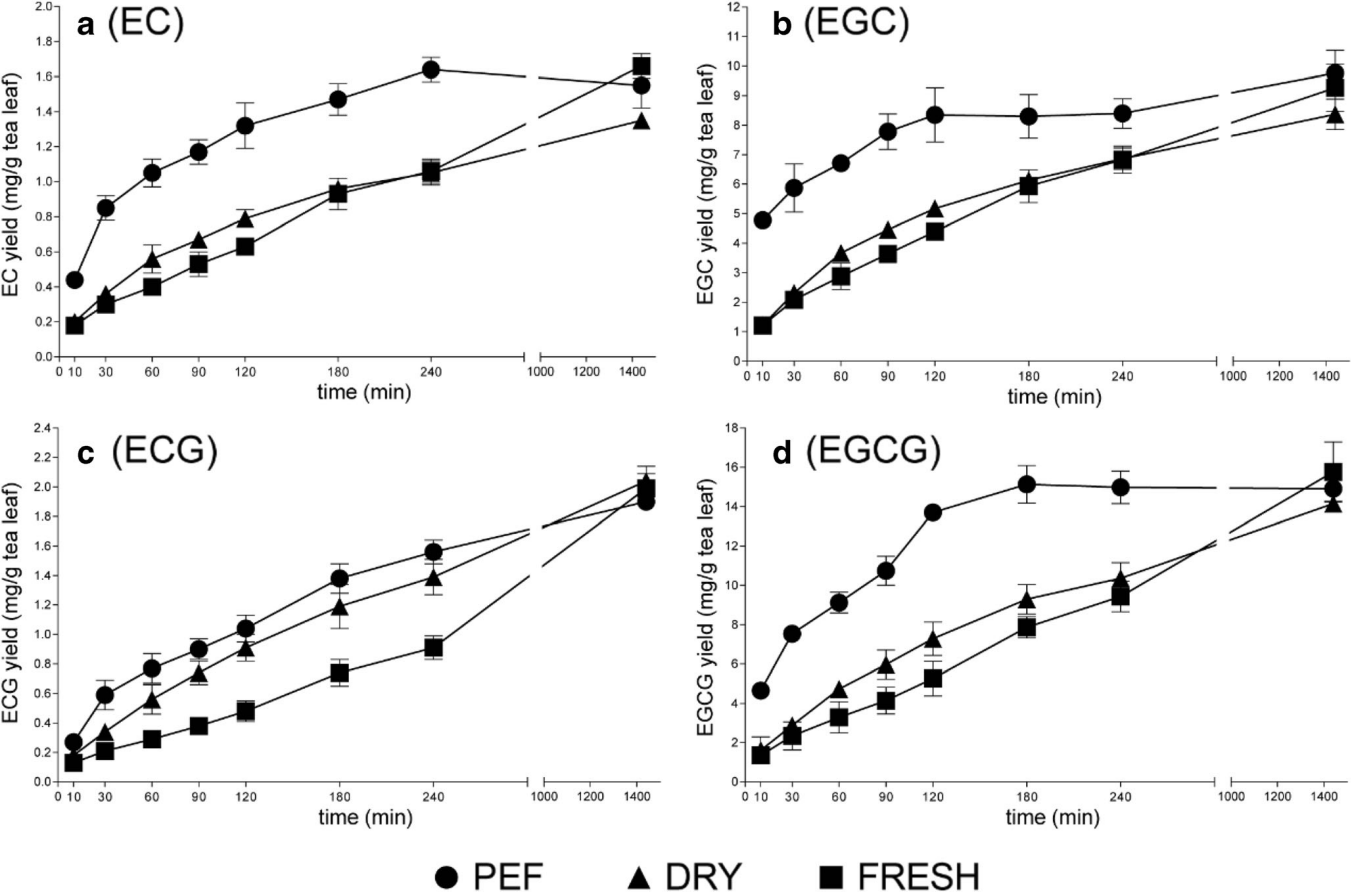
The extraction kinetics of EC (**a**), EGC (**b**), ECG (**c**), and EGCG (**d**) from fresh tea leaves pre-treated with PEF, oven-drying, and untreated. Values represent the means ± SD of triplicate measurements

In order to evaluate the extraction efficiency of PEF pre-treatment, the release of phenolics from tea leaves was studied along the extraction time. The Folin-Ciocalteu assay used in the optimization of the PEF conditions mainly determines the number of aromatic rings, and therefore, its accuracy and specificity is relatively low. Thus, LC/MS targeting the four primary catechins, including EC, EGC, ECG, and EGCG, was employed to better monitor the dynamics of phenolics during the extraction. A 24-h kinetic experiment of the catechins extracted with acetone (50%, *w*/*w*) was conducted after the PEF pre-treatment (*E* = 1.00 kV/cm; *n* = 100; *τ* = 100 μs and △*t* = 5 s). The fresh and oven-dried samples with the same acetone extraction procedures were used for comparison. To assess the residual polyphenols after the 24-h extraction, an additional 2-h extraction with fresh acetone (50%, *w*/*w*) was performed. LC/MS analysis indicated that the residual contents of EC, EGC, ECG, and EGCG in the subsequent 2-h extraction were less than 2% of those in the extracts at 24 h (data not shown). Thus, the 24-h extraction was considered as a complete extraction for the four catechins in both PEF-treated and PEF-untreated samples, and gave similar yields for all treatments. The extraction yields of each catechin at certain time points are plotted against the irrespective extraction time, as presented in Fig. 4. It shows that notably the extraction yield of the PEF-treated samples after the first few hours is significantly larger compared to dried and fresh leaves. After 24-h extraction, all pre-treatments result in a similar maximal yield, which were approximately 1.7, 9.8, 2.0, and 15.8 mg/g fresh tea leaf for EC, EGC, ECG, and EGCG, respectively. These values were generally in line with the catechin contents reported by Rahim et al. (2014).

In addition, extraction kinetics were modeled assuming first-order kinetics to analyze the apparent extraction rate of the four catechins: $\frac{d{C}_t}{dt}={k}_{obs}\left({C}_e-{C}_t\right)$, where *C*_*t*_ is the concentration of catechin at time *t*, *C*_*e*_ is the equilibrium concentration of catechin, and *k*_obs_ is the first-order observed extraction rate constant. If we assume that the initial concentration of catechin is zero at time *t* = 0, then *k*_obs_ can be calculated as the slope of the linear regression of $\ln \left(\frac{C_e-{C}_t}{C_e}\right)=-{k}_{obs}\ t$ (Spiro and Jago 1982). Data outside the linear range was not used for the regression. The catechin extraction rate constants of PEF-treated, oven-dried, and fresh tea leaves are summarized in Table 1. As expected, fresh tea leaves without any pre-treatment had the lowest catechin extraction rate constant compared to the PEF-treated or oven-dried samples. The low extraction rate constant observed for untreated tea leaves was probably due to the integrity of cell membrane. PEF-assisted extraction appeared more promising for extraction of tea polyphenols, with an approximately 3.6 times higher extraction rate constant than that for untreated leaves. The improvement of extraction yield was attributed to the permeabilization effect of PEF on tea leaf cells. It has also been reported that the extraction of polyphenols from orange peel (Luengo et al. 2013), grape skin (López et al. 2008), and flaxseed hulls (Boussetta et al. 2014) were significantly improved after PEF treatment. Moreover, higher extraction rate constants of catechins (2.3-fold higher) were also observed for PEF-treated samples compared to conventional oven-dried samples.

**Table 1:** The acetone extraction rate constants (*k*_obs_, h^−1^) of PEF treated, oven-dried, and fresh tea leaves

	PEF	Dry	Fresh
EC	0.82	0.41	0.26
EGC	1.06	0.45	0.33
ECG	0.44	0.30	0.15
EGCG	1.12	0.35	0.22

### Phenolic Profiles of Extracts

**Fig. 5:**
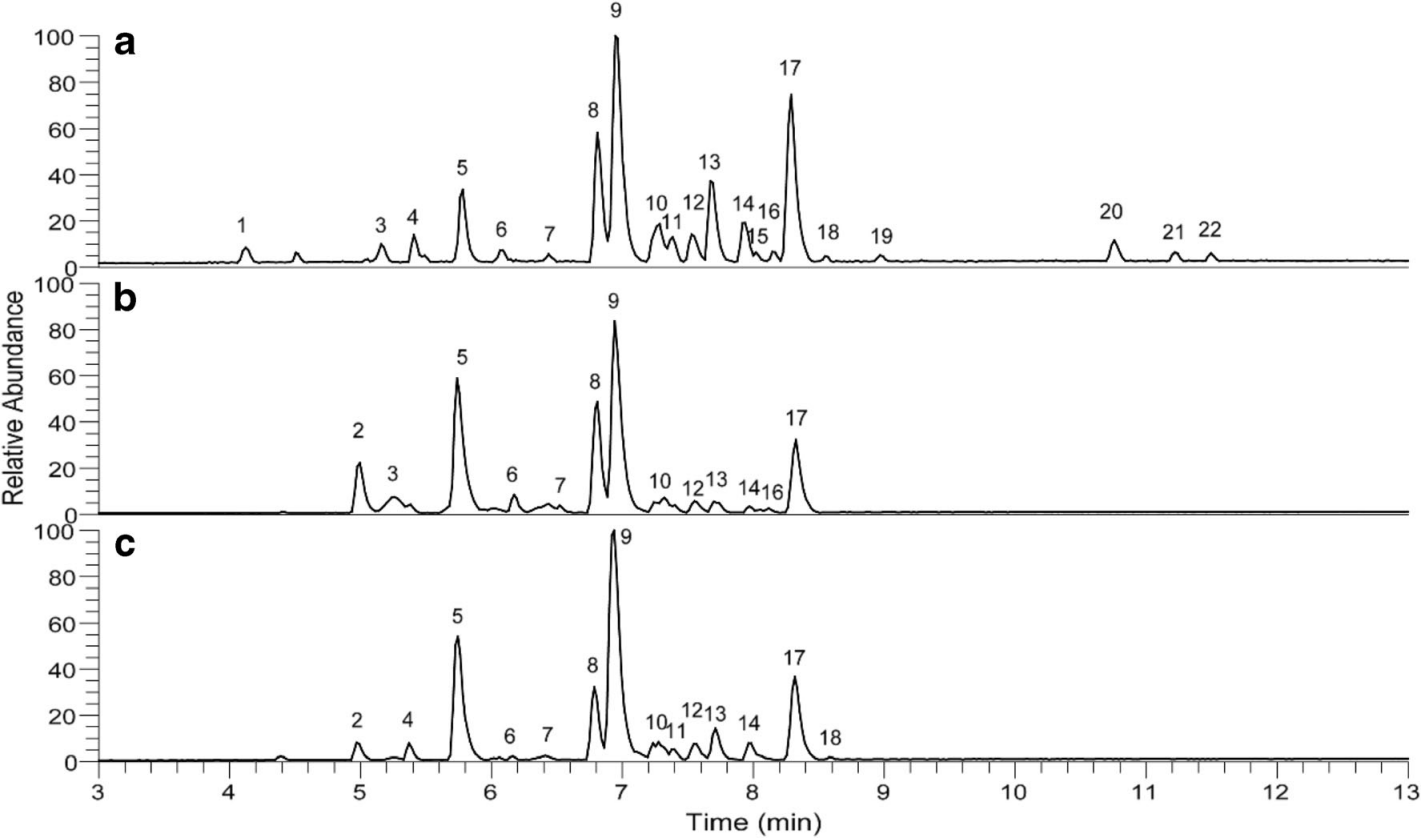
RP-UHPLC-MS base peak chromatogram (*m*/*z* 150–1500, negative ionization mode) of the extracts from PEF-treated (**a**), fresh (**b**), and oven-dried (**c**) tea leaves

It has not yet been reported whether the application of an external electric field would modify the profile of tea polyphenols. Thus, RP-UHPLC-MS was further utilized to compare the phenolic profile of the PEF-treated tea leaf (*E* = 1.00 kV/cm; *n* = 100; *τ* = 100 μs and △*t* = 5 s), followed with a 2-h acetone extraction, with fresh and oven-dried samples with the same acetone extraction procedure. The chromatograms obtained are presented in Fig. 5. Peaks shown in the chromatograms were annotated based on the comparison of MS/MS fragmentation and *λ*_max_ with published data (Lin et al. 2008; Verloop et al. 2016; García-Villalba et al. 2017; Wu et al. 2012), as listed in Table 2. A total of 22 components were tentatively annotated, of which EC (peak no. 8), EGC (peak no. 5), ECG (peak no. 17), and EGCG (peak no. 9) were recognized as the most abundant native catechins. Other components, including 10 flavanols and 8 flavonols, were also detected from LC/MS analysis. Due to their relatively low abundance, some of these compounds did not clearly show in chromatograms. In general, the three phenolic profiles were similar and in line with our previous observations (Verloop et al. 2016; Liu et al. 2016).

**Table 2:** Phenolic compounds tentatively identified by RP-UHPLC–MS from PEF treated, oven-dried and fresh tea leaves

Peak no.^a^	Retention time (min)	*λ*_max_ (nm)^b^	[M-H]^−^ (*m*/*z*)	MS^2^ fragment (*m*/*z*)	Tentative identification	Formula
1	4.12	270; 380	625	581; 443; 563	Dehydrotheasinensin C	C_27_ H_30_ O_17_
2	4.95	270	305	221; 179; 261; 165; 287	Gallocatechin	C_15_ H_14_ O_7_
3	5.16	270	759	607; 589; 451	Theacitrin-3-gallate	C_37_ H_28_ O_18_
4	5.41	270	467	305; 329; 423; 261	Catechin-hexoside	C_21_ H_24_ O_12_
5	5.78	270	305	221; 261; 179; 165; 125; 287	Epigallocatechin	C_15_ H_14_ O_7_
6	6.09	271	289	245; 205; 179	Catechin	C_15_ H_14_ O_6_
7	6.44	270	593	473; 503; 353; 383	Kaempferol-rhamnoside-hexoside	C_30_ H_26_ O_13_
8	6.81	272; 329	289	245; 204; 179	Epicatechin	C_15_ H_14_ O_6_
9	6.94	273	457	169; 331; 305	Epigallocatechin-gallate	C_22_ H_18_ O_11_
10	7.28	351; 266	771	301; 609	Rutin-hexoside	C_33_ H_40_ O_21_
11	7.38	351; 266	771	301; 609	Rutin-hexoside	C_33_ H_40_ O_21_
12	7.53	270	577	413; 293	Procyanidin B2	C_27_ H_30_ O_14_
13	7.67	270	609	301	Rutin (quercetin rutinoside)	C_27_ H_30_ O_16_
14	7.94	270; 355	755	285	Quercetin-rhamnosyl-rutinoside	C_33_ H_40_ O_20_
15	8.02	270; 375	463	301	Quercetin hexoside	C_21_ H_20_ O_12_
16	8.15	277	755	285	Quercetin-rhamnosyl-rutinoside	C_33_ H_40_ O_20_
17	8.29	274	441	289; 331; 169	Epicatechin-gallate	C_22_ H_18_ O_10_
18	8.55	273; 375	447	284; 327	Kaempferol-hexoside	C_21_ H_20_ O_11_
19	8.97	273; 375	759	589; 607	Theacitrin-3′-gallate	C_37_ H_28_ O_18_
20	10.76	375	563	545; 407; 519; 379; 425	Theaflavin	C_29_ H_24_ O_12_
21	11.22	375	715	527; 563; 545; 577; 501	Theaflavin-3-gallate	C_36_ H_28_ O_16_
22	11.50	375	715	563; 545; 407; 577; 697	Theaflavin-3′-gallate	C_36_ H_28_ O_16_

^a^Peak numbers were assigned from the chromatograms in Fig. 5

^b^Only *λ*_max_ above 250 nm are shown

It is noteworthy that three minor oxidation products of catechins, including theaflavin (peak no. 20), theaflavin-3-gallate (peak no. 21), and theaflavin-3′-gallate (peak no. 22), were tentatively annotated in PEF-treated samples, which were not presented in fresh or oven-dried samples. These compounds were likely formed upon catalysis by endogenous oxidative enzymes in fresh tea leaves during permeabilization by the PEF treatment. Thus, oxidation of phenolics should be considered when applying PEF treatment in aqueous environment, because permeabilization by PEF will facilitate contact between phenolics, oxygen, and endogenous oxidative enzymes, even though these enzymes will be inactivated by the subsequent acetone extraction. Shortening of the PEF treatment time or addition of reducing agents during PEF treatment might inhibit such oxidation.

Generally, in addition to minor oxidation, the moderate PEF treatments conducted in this study did not affect the composition of tea polyphenols. Similar results were obtained by Puértolas et al., who reported that the application of a PEF treatment to purple-fleshed potato before extraction did not affect the profile of anthocyanin, but only obtained more anthocyanins (Puértolas et al. 2013). Such findings could be related to the permeabilization of cells by PEF treatment. Furthermore, the phenolic profiles at different pulsed electric field strength (*E* = 0.75 kV/cm, 1.00 kV/cm, and 1.25 kV/cm) were also compared. In general, similar phenolic profiles were obtained (data not shown).

### Tissue Morphology Analysis

**Fig. 6:**
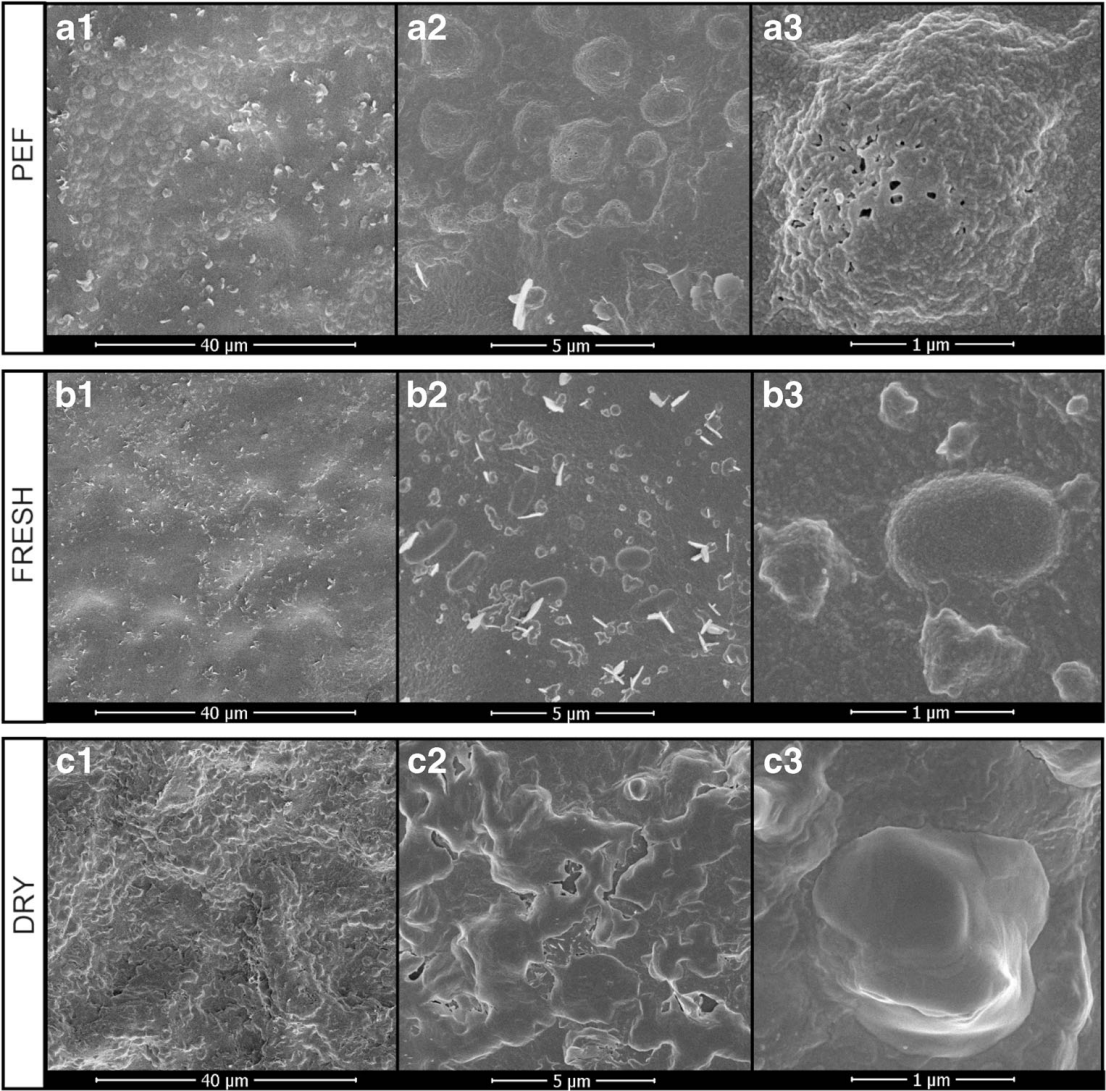
SEM images of upper surface of tea leaf tissue pre-treated with PEF (A1–A3), untreated (B1–B3), and oven-drying (C1–C3), with the magnifications of × 2000 (A1, B1, C1), × 10,000 (A2, B2, C2), and × 50,000 (A3, B3, C3)

In addition to the extraction yield and phenolic composition assays, the micromorphology changes of tea leaf tissues after PEF treatment were also studied. SEM studies carried out on the surface of PEF-treated and PEF-untreated (fresh and oven-dried) tea leaf tissues displayed remarkable differences, as shown in Fig. 6. The fresh untreated tea leaf samples preserved their integrity, with relatively smoother cell surface. Some regular pellets and epicuticular wax crystals diffused on the cell surface, but no pores were clearly visible (Fig. 6(B1–B3)). After PEF treatment, unevenly distributed pores could be clearly observed on the surface (Fig. 6(A3)), with a diameter of 50–100 nm. The extended surface of tea leaf tissue as a conductor gives the perpendicular field for charge to accumulate on either side, which leads to a high local electric field and a local breakdown, thus releasing enough energy to puncture a hole. The occurrence of pores after PEF treatment was previously descripted by other researchers. For example, it was observed by Koubaa et al. that the cells of *Opuntia stricta* fruit peels presented pores on their surfaces after PEF treatment (20 kV/cm, 50 pulses) (Koubaa et al. 2016).

In addition to the electroporation, a considerable number of protuberances with highly corrugated surface were formed after PEF treatment (Fig. 6(A1–A3)). The impact of the external electric field might be the cause of the corrugation. It is also worthy of note that the pores seem to be mainly located on these protuberances (Fig. 6(A2, A3)). The increase of the porosity and formation of protuberances on the tea leaf cells may improve the capacity of penetration of the acetone into the cells and the diffusion of phenolics through the cell membranes, which explains the improvement of polyphenol extraction observed.

For oven-dried samples, significant roughness and plication emerged on the surface of tea leaf (Fig. 6(C1, C2)). Due to the loss of water during oven-drying, cell shrinkage and distortion can occur, and thus, large cavities can be formed (Drosou et al. 2015). However, unlike PEF-treated samples, the protuberances seemed to be smooth without significant cracks or pores (Fig. 6(C3)), which might indicate that the structural disorder effect of drying occurred at micron-scale, whereas for PEF, it was at nano-scale phenomenon. This might also explain why PEF treatment exerts better permeabilization than conventional thermal dehydration. The results presented here provided visual comparison of the different impacts of PEF treatment and thermal dehydration process on the micromorphology of tea leaf tissues.

In addition, it was also found that similar protuberances and pores were observed among different electric field intensities (figures not shown). It was difficult to relate the number or size of protuberances and pores to the electric field intensity.

## Conclusions

The results obtained in this study demonstrated that compared to the conventional thermal drying, PEF treatment on fresh tea leaves before extraction had the advantages of improving the extraction yield, shortening the extraction time, lowering the energy input, and controlling the operational temperature, but without significantly altering the phenolic profiles. Thus, PEF pre-treatment can be used to replace the time and energy-intense drying procedure. The improved extraction efficiency observed in this study was due to the application of the external electric field that induced the formation of large numbers of pores and protuberances on the surface of tea leaf tissues, thus improving the permeability and facilitating the release of phenolics.
